# Identifying genetic risk factors driving cerebral amyloid angiopathy using novel mouse models

**DOI:** 10.1002/alz.091446

**Published:** 2025-01-03

**Authors:** Olivia J Marola, Kristen D Onos, Cory Diemler, Kelly J Keezer, Michael Sasner, Gareth R Howell, the IU/JAX/PITT MODEL‐AD Center

**Affiliations:** ^1^ The Jackson Laboratory, Bar Harbor, ME USA

## Abstract

**Background:**

Cerebral amyloid angiopathy (CAA) co‐occurs with neurodegeneration in Alzheimer’s disease (AD). CAA is absent in many AD mouse models, rendering CAA difficult to study. Previous work has shown wild‐derived WSB/EiJ (WSB) mice over‐expressing *APP/PS1* had increased CAA, and thus may be useful in investigating CAA‐causing mechanisms. Here, genetic backgrounds with CAA susceptibility (WSB.*APP/PS1*) and resilience (B6.*APP/PS1*) are leveraged to map CAA‐susceptibility loci.

Furthermore, blood brain barrier (BBB) compromise has been hypothesized to impair amyloid clearance and promote vascular deposition. Endothelial TIE2 deactivation was shown to weaken BBB integrity in other models, and therefore may drive CAA. TIE2 activation (pTIE2) is inhibited by endogenous antagonists ANGPT2 and PTPRB, which can be manipulated to bolster pTIE2. Here, the efficacy of pTIE2 elevation in preventing CAA in AD is investigated.

**Methods:**

To determine the onset and therapeutic window of CAA, WSB.*APP/PS1* mice (n≥3/sex) were aged to 4, 6, 8, 14, and 18M. CAA was assessed using immunohistochemistry (X34+ plaques within CD31+ vessels). To map CAA‐susceptibility loci, B6.*APP/PS1* and WSB mice were crossed, generating BXW.*APP/PS1* (F1) offspring. To determine haplosufficiency of these alleles, CAA was evaluated in BXW.*APP/PS1* mice (n = 6/sex). F1 mice were intercrossed to generate heterogeneous F2 progeny (n = 50/sex), which were assessed for CAA.

To elevate pTIE2 in models of CAA/AD, *Angpt2* heterozygous *(Angpt2^±^)* and *Ptprb* loss‐of‐function (*Ptprb* ^D57N^) alleles were bred to generate CAA‐susceptible mice with TIE2 modulation. Western blot analysis is employed to confirm elevated pTIE2, and CAA amelioration will be evaluated.

**Results:**

WSB.*APP/PS1* mice developed significant CAA by 8M. CAA was significantly more advanced in BXW.*APP/PS1* compared to founder strains at 8M, and F2 mice had varying CAA severity. Late‐onset AD mice (LOAD2, JAX #033867) with *Ptprb^D57N/D57N^* exhibited pTIE2 elevation at 12M.

**Conclusions:**

Robust BXW.*APP/PS1* CAA suggests heterozygous loci promote CAA. F2 CAA variability suggests CAA‐susceptibility loci are likely mappable. F2 SNP analysis and R/qtl2 software is being employed to identify candidate loci corresponding with CAA. Furthermore, BXW.*APP/PS1* is a novel CAA model to study the therapeutic potential of pTIE2 enhancement. To this end, BXW.*Angpt2^±^APP/PS1* and BXW.*Ptprb^+/D57N^APP/PS1* mice are aging and will be evaluated for pTIE2 elevation and CAA attenuation.

